# Performance on sprint, agility and jump tests have moderate to strong correlations in youth football players but performance tests are weakly correlated to neuromuscular control tests

**DOI:** 10.1007/s00167-020-06302-z

**Published:** 2020-10-08

**Authors:** Sofi Sonesson, Hanna Lindblom, Martin Hägglund

**Affiliations:** 1 Unit of Physiotherapy, Department of Health, Medicine and Caring Sciences, Linköping University, Campus US, S-581 83 Linköping, Sweden; 2 Sport Without Injury ProgrammE (SWIPE), Department of Health, Medicine and Caring Sciences, Linköping University, Linköping, Sweden

**Keywords:** Sprint test, Agility test, Jump test, Adolescents, Male, Female, Soccer

## Abstract

**Purpose:**

This study aimed at evaluating the correlation between seven different performance tests and two neuromuscular control tests in youth football players and to evaluate the influence of sex and age groups on test results.

**Methods:**

One-hundred and fifteen football players (66 boys, 49 girls) mean age 14 ± 0.7 (range 13–16) years from youth teams were tested at the start of the second half of the competitive season. A test battery including agility t-test, 505 agility test, single-leg hop for distance test, side-hop test, countermovement jump test, 10-m sprint test, 20-m sprint test, tuck jump assessment (TJA) and drop vertical jump (DVJ) was completed.

**Results:**

Correlations between the seven different performance tests of agility, jump and sprint ability were generally moderate to strong (r = 0.534–0.971). DVJ did not correlate with the performance tests (rho = 0.004 to  –  0.101) or with TJA total score (rho = 0.127). There were weak to moderate correlations between TJA total score and the performance tests (*r* =  – 0.323–0.523). Boys performed better than girls in all performance tests (*p* < *0.001*) and in TJA total score (*p* = *0.002*). In boys, older players performed better than younger players in the majority of the tests, while there was no clear age influence among girls.

**Conclusion:**

Sprint performance was moderately to strongly correlated with agility and jump performance, and performance tests were weakly to moderately correlated to TJA, while DVJ did not correlate with the other tests. Boys performed better than girls on performance tests and TJA. An age effect on performance was evident in boys but not in girls.

**Level of evidence:**

Level IV

**Trial registration:**

Clinical Trials gov identifier: NCT03251404

**Electronic supplementary material:**

The online version of this article (10.1007/s00167-020-06302-z) contains supplementary material, which is available to authorized users.

## Introduction

Performance tests are often used to evaluate agility, jump and sprint performance in athletes, which are important physical attributes for successful participation in team sports [31, 33]. Power and speed abilities are important physical components in the game [6, 10, 12, 37] and high-intensity endurance capacity in youth players may predict future career progression [7]. Physical demands and capacity vary between individual players and may depend on sex, level, playing style and position in the team [3, 4, 6, 39]. In youth football, physical performance is generally increased in older age groups, although some studies report a plateau from 15 years of age [21, 24, 40]. Differences between age groups may be attributed to maturation or different training status.

A battery of field-based tests has been recommended to assess various aspects of performance and neuromuscular control in male youth football players [36]. Screening tests for assessment of neuromuscular control may capture biomechanical parameters associated with increased risk of injury [16, 17] and reveal adaptations in movement patterns after injury prevention exercise programs [26, 29, 30]; however, it is debated whether screening tests can predict injury [18, 23]. Football players [22], particularly female youth players [13], have a high risk of ACL injury. Reduced neuromuscular control and increased knee valgus loading are associated with increased risk of ACL injury [16], and this movement pattern is common in youth female players [32]. Different tests are used to assess different key components of physical performance and neuromuscular control [36] but the correlation between various aspects of performance and neuromuscular control in youth football players is unclear. It is a challenge to select tests to be included in a test battery to gain maximum knowledge from a minimum number of tests and, therefore, there is a need to increase the knowledge on the relationship between tests. The purposes were (1) to evaluate the correlation between seven different performance tests and two neuromuscular control tests in youth football players, and (2) to evaluate the influence of sex and age groups on test results.

## Material and methods

The study was approved by the Swedish Ethical Review Authority: Dnr 2017/294–31. All players and their guardians signed an informed consent prior to participation. One-hundred and fifteen football (soccer) players (66 boys, 49 girls) mean age 14 ± 0.7 (range 13–16) years from eight youth teams were tested. All teams had scheduled football training at least two sessions per week. Characteristics of the players are displayed in Table 1. All players were informed that to take part in testing they should be able to participate with maximum effort. The study was carried out in Östergötland, Sweden. Testing was carried out at the start of the second half of the competitive season after the school summer break in August–September 2017. No players reported injuries at the time of testing, and all were participating regularly in football training.

**Table 1:** Player characteristics

Variable	Male (*n* = 66)	Female (*n* = 49)	Total (*N* = 115)
Age, years, mean ± SD (range)	14.0 ± 0.6 (13–15)	14.0 ± 0.8 (13–16)	14.0 ± 0.7 (13–16)
Height, cm, mean ± SD	167 ± 9	164 ± 7	166 ± 9
Body mass, kg, mean ± SD	56 ± 11	55 ± 9	55 ± 10
Years of football experience, mean ± SD	7.6 ± 2.2	7.1 ± 1.8	7.4 ± 2.0
Football exposure, mean ± SD^1^	4.3 ± 1.4	4.3 ± 1.1	4.3 ± 1.3
Other sport exposure, mean ± SD^1^	2.3 ± 1.7	1.5 ± 1.2	2.0 ± 1.6
Football profile at school (*n*)	29	11	40

^1^Number of training sessions and matches each week

### Assessments

Players were asked to refrain from physically exhausting training on the day before testing. All players were recommended to wear tight shorts, t-shirt, short socks and indoor shoes. Due to their preference, five players performed the tests barefoot. Two sports physiotherapists and two physiotherapy students served as test leaders. They used standardized instructions and demonstrated the performance of each test before the players made the practice trials. Testing was carried out for one team at a time and it took about 2 h to complete the test battery for the whole team. Between trials other players were tested to make sure the players recovered between the trials and were able to perform at their maximum capacity. Before testing all players performed a standardized warm-up for 5 min consisting of running and agility drills. The test battery included nine tests performed in the same order for all players: drop vertical jump (DVJ), agility t-test, single-leg hop for distance test, 505 agility test, side-hop test, 10- and 20-m sprint test, tuck jump assessment (TJA) and countermovement jump test. Agility tests, jump tests and sprint tests were used to assess performance and DVJ and TJA were used to assess neuromuscular control (online appendix).

### Equipment

Timing gates with photoelectric cells (MuscleLab 4010, Ergotest Technology a.s., Norway) were used for the agility and sprint tests and an infrared contact mat (MuscleLab 4010, Ergotest Technology a.s., Norway) was used during the test for vertical jump height.

Two GoPro Hero5 cameras that started and stopped synchronically were used for the neuromuscular control tests and for the side hop test. Cameras were positioned in the frontal plane (TJA and DVJ) and the sagittal plane (TJA) and films were used for post-test assessment.

### Statistical analyses

Analyses were performed with SPSS (IBM SPSS Statistics for Windows, Version 25. Armonk, NY: IBM Corp.), and all analyses were two-sided, with the significance level set at *p* < 0.05. Pearson’s product-moment correlation coefficient (*r*) was used to analyze linear correlations between all tests except DVJ where Spearman´s rank correlation coefficient (*rho*) was used. Strength of correlation coefficients was interpreted as follows: negligible ≤ 0.30, weak 0.31–0.50, moderate 0.51–0.70, or strong > 0.71. Bias corrected and accelerated (BCa) 95% confidence intervals was used. One-way analysis of variance (ANOVA) with additional pairwise contrasts was used to estimate between age group effects, on all seven performance tests and number of jumps during TJA. Effect size measures, partial omega-squared (*ω*_*p*_^*2*^) was calculated for all main effects between age groups, and Cohen’s *d* was calculated for all pairwise contrasts. Kruskal–Wallis *H* test with additional Mann–Whitney *U*-tests for pairwise comparisons was used to compare between age group differences in mean rank of TJA. Effect size measures, partial eta-squared (*η*_*p*_^*2*^), and Cohen’s *d* were calculated as main and pairwise effects between age groups, respectively. Independent samples t-tests were used to estimate between sex effects on all seven performance tests and number of jumps during TJA. Mann–Whitney *U*-test was used to compare differences in mean rank of TJA between sexes. Cohen’s *d* was calculated for all between-sex effects. Associations between DVJ, sex and age groups were assessed with Fisher’s exact tests. Calculation of Cramer’s V was used to measure the strength of the associations. The following limits were used for interpretation of effect size measures: *ω*_*p*_^*2*^ ≥ 0.01 = small effect, *ω*_*p*_^*2*^ ≥ 0.06 = medium effect, *ω*_*p*_^*2*^ ≥ 0.14 = large effect; *d* ≥ 0.2 = small effect, *d* ≥ 0.5 = medium effect, *d* ≥ 0.8 = large effect; *η*_*p*_^*2*^ ≥ 0.01 = small effect, *η*_*p*_^*2*^ ≥ 0.06 = medium effect, *η*_*p*_^*2*^ ≥ 0.14 = large effect.

## Results

### Correlations between tests

Correlations between different performance tests were moderate to strong (r = 0.534–0.971), except between 505-agility test and side hop (*r* =  – 0.487). Agility t-test was strongly correlated to 505-agility test (*r* = 0.775). Correlations between single-leg hop for distance, side hop and CMJ were moderate (*r *= 0.534–0.694). 10 m sprint test and 20 m sprint test were strongly correlated (*r* = 0.971). There were weak to moderate correlations between TJA total score and the seven performance tests (*r* =  – 0.329–0.523). Number of jumps during TJA did not correlate with any variable (*r* = 0.017–0.153). The DVJ did not correlate with the seven performance tests (rho = 0.012 to  – 0.064) nor with TJA total score (rho = 0.149) (Table 2).

**Table 2:** Correlation between tests assessing performance or neuromuscular control

		Agility t-test (s)	505 agility test (s)	Hop for distance (cm)	Side hop (n)	CMJ (cm)	10 m sprint (s)	20 m sprint (s)	TJA total score	TJA number of jumps	DVJ
		(*n* = 115)	(*n* = 115)	(*n* = 115)	(*n* = 115)	(*n* = 115)	(*n* = 115)	(*n* = 115)	(*n* = 114)	(*n* = 115)	(*n* = 115)
Agility t-test (s)		1									
505 agility test (s)	*r*	0.775**	1								
	BCa 95% CI	(0.674–0.857)									
Hop for distance (cm)	*r*	– 0.697**	– 0.648**	1							
	BCa 95% CI	(– 0.784 to –0.592)	(– 0.745 to –0.527)								
Side hop (*n*)	*r*	– 0.619**	– 0.487**	0.640**	1						
	BCa 95% CI	(– 0.725 to –0.481)	(– 0.615 to –0.332)	(0.504–0.739)							
CMJ (cm)	*r*	– 0.658**	– 0.601**	0.694**	0.534**	1					
	BCa 95% CI	(– 0.742 to – 0.56)	(– 0.706 to –0.472)	(0.569–0.793)	(0.390–0.655)						
10-m sprint (s)	*r*	0.796**	0.769**	– 0.759**	– 0.602**	– 0.776**	1				
	BCa 95% CI	(0.704–0.862)	(0.668–0.846)	(– 0.826 to – 0.667)	(– 0.705 to –0.466)	(– 0.830 to –0.705)					
20-m sprint (s)	*r*	0.814**	0.781**	– 0.743**	– 0.609**	– 0.760**	0.971**	1			
	BCa 95% CI	(0.735–0.873)	(0.677–0.861)	(– 0.812 to –0.652)	(– 0.714 to – 0.475)	(– 0.828 to –0.670)	(0.958–0.980)				
TJA total score	*r*	0.425**	0.329**	– 0.381**	– 0.345**	– 0.501**	0.523**	0.518**	1		
	BCa 95% CI	(0.274–0.560)	(0.127–0.484)	(– 0.545 to – 0.195)	(– 0.513 to – 0.161)	(– 0.632 to – 0.355)	(0.369–0.644)	(0.356–0.639)			
TJA number of jumps	*r*	– 0.069	– 0.079	0.017	0.153	– 0.100	– 0.022	– 0.025	– 0.018	1	
	BCa 95% CI	(– 0.279–0.147)	(– 0.266–0.141)	(– 0.196–0.216)	(– 0.063–0.344)	(– 0.284–0.073)	(– 0.214–0.182)	(– 0.216–0.172)	(– 0.184–0.159)		
DVJ	*rho*	0.012	0.055	– 0.055	– 0.055	– 0.064	0.024	0.015	0.149	– 0.057	1
	BCa 95% CI	(– 0.150–0.188)	(–0.133–0.237)	(–0.235–0.137)	(– 0.275–0.152)	(– 0.241–0.114)	(– 0.154–0.184)	(– 0.175–0.184)	(– 0.043–0.327)	(– 0.234–0.131)	

Hop for distance, single-leg hop for distance, *CMJ* countermovement jump, *TJA* tuck jump assessment, *DVJ* drop vertical jump, *BCa* bias-corrected and accelerated, *CI* confidence interval

^*^p < 0.05 **p < 0.01 ***p < 0.001

### Influence of sex and age on test results

Boys performed better than girls on all performance tests (*p* < *0.001*) and in TJA total score (*p* = *0.002*, small to large effect sizes; Cohen’s *d* 0.655–1.501) (Table 3). There was no sex difference in DVJ (Table 4). In boys, older players performed better than younger players in the majority of the performance tests and in TJA (*p* = 0.036–*p* < *0.001*, medium to large effect sizes; *ω*_*p*_^2^ 0.07–0.265) (Table 3). The neuromuscular control during DVJ differed between age groups in boys, where the majority of the 13-year-old players had good knee control and the majority of the 15- to 16-year-old players had reduced knee control (*p* = *0.006*, large effect size; Cramer´s V 0.336) (Table 4). In girls, the agility tests differed between age groups, where the youngest players performed worse (*p* = *0.013*, *p* = *0.002*, medium to large effect sizes; *ω*_*p*_^2^ 0.135–0.202), while there was no age influence in the other tests (Tables 3 and 4).

**Table 3:** Age and sex differences in tests assessing performance or neuromuscular control

		Age groups				Between age groups effects		Pairwise contrasts					
		13 years (a)	14 years (b)	15-16 years (c)	Total			a–b		a–c		b–c	
		Mean ± SD	Mean ± SD	Mean ± SD	Mean ± SD	p-value	*ω*_*p*_^*2*^	*p*-value	*d*	*p*-value	*d*	*p*-value	*d*
Agility t-test (s)	Male	12.8 ± 0.9	11.8 ± 0.6	11.5 ± 0.7	11.9 ± 0.8	< 0.001	0.244	< 0.001	1.031	< 0.001	1.160	n.s	0.398
	Female	13.5 ± 0.7	12.7 ± 0.6	12.8 ± 0.8	12.9 ± 0.8	0.002	0.202	< 0.001	1.091	0.012	0.775	n.s	0.184
	Total	13.2 ± 0.8	12.1 ± 0.7	12.1 ± 1.0	12.4 ± 0.9	< 0.001	0.213	< 0.001	1.043	< 0.001	0.876	n.s	0.004
Between-sex effects	*p*-value	0.040	< 0.001	< 0.001	< 0.001								
	*d*	0.919	1.305	1.734	1.289								
505 agility test (s)	Male	2.7 ± 0.2	2.6 ± 0.2	2.5 ± 0.1	2.6 ± 0.2	0.036	0.072	n.s	0.474	0.010	0.667	n.s	0.361
	Female	2.9 ± 0.1	2.8 ± 0.1	2.8 ± 0.1	2.8 ± 0.1	0.013	0.135	0.004	0.905	n.s	0.429	n.s	0.396
	Total	2.8 ± 0.2	2.7 ± 0.2	2.7 ± 0.2	2.7 ± 0.2	0.002	0.093	< 0.001	0.681	0.005	0.543	n.s	0.033
Between-sex effects	*p*-value	0.020	< 0.001	< 0.001	< 0.001								
	*d*	1.060	0.939	2.286	1.233								
Single-leg hop for distance (cm)	Male	127.1 ± 18.4	142.9 ± 16.5	147.0 ± 16.8	141.3 ± 17.7	0.015	0.097	0.009	0.674	0.007	0.707	n.s	0.192
	Female	129.8 ± 12.3	127.5 ± 12.4	120.3 ± 13.9	126.4 ± 13.0	n.s	0.037	n.s	0.154	n.s	0.548	n.s	0.471
	Total	128.6 ± 14.9	137.4 ± 16.8	134.2 ± 20.3	134.9 ± 17.5	n.s	0.021	0.038	0.397	n.s	0.210	n.s	0.150
Between-sex effects	*p*-value	n.s	< 0.001	< 0.001	< 0.001								
	*d*	0.178	1.015	1.722	0.942								
Side hop (n)	Male	27.7 ± 10.7	36.5 ± 12.0	39.3 ± 11.6	35.7 ± 12.1	n.s	0.059	0.037	0.538	0.021	0.594	n.s	0.193
	Female	29.5 ± 8.1	28.7 ± 11.9	25.7 ± 11.2	28.2 ± 10.7	n.s	-0.024	n.s	0.058	n.s	0.258	n.s	0.236
	Total	28.7 ± 9.1	33.7 ± 12.4	32.8 ± 13.1	32.5 ± 12.1	n.s	0.009	n.s	0.326	n.s	0.222	n.s	0.063
Between-sex effects	*p*-value	n.s	0.013	0.006	< 0.001								
	*d*	0.189	0.650	1.201	0.655								
CMJ (cm)	Male	22.7 ± 4.9	24.4 ± 4	26.6 ± 3.8	24.6 ± 4.2	n.s	0.048	n.s	0.297	0.028	0.566	n.s	0.423
	Female	21.1 ± 2.8	20.4 ± 4.2	17.8 ± 4.1	19.9 ± 4	n.s	0.065	n.s	0.156	0.036	0.637	n.s	0.570
	Total	21.8 ± 3.9	23 ± 4.5	22.4 ± 6	22.6 ± 4.7	n.s	-0.007	n.s	0.196	n.s	0.078	n.s	0.105
Between-sex effects	*p*-value	n.s	< 0.001	< 0.001	< 0.001								
	*d*	0.430	0.995	2.244	1.138								
10 m sprint (s)	Male	2.0 ± 0.1	1.9 ± 0.1	1.8 ± 0.1	1.9 ± 0.1	< 0.001	0.265	< 0.001	0.945	< 0.001	1.265	0.014	0.634
	Female	2.0 ± 0.1	2.0 ± 0.1	2.0 ± 0.1	2.0 ± 0.1	n.s	0.021	n.s	0.405	n.s	0.037	n.s	0.436
	Total	2.0 ± 0.1	1.9 ± 0.1	1.9 ± 0.2	1.9 ± 0.1	< 0.001	0.101	< 0.001	0.692	0.002	0.607	n.s	0.034
Between-sex effects	*p*-value	n.s	< 0.001	< 0.001	< 0.001								
	*d*	0.194	1.150	2.338	1.180								
20 m sprint (s)	Male	3.6 ± 0.2	3.4 ± 0.2	3.2 ± 0.2	3.4 ± 0.2	< 0.001	0.260	< 0.001	0.989	< 0.001	1.239	0.033	0.550
	Female	3.7 ± 0.2	3.5 ± 0.2	3.6 ± 0.2	3.6 ± 0.2	n.s	0.069	0.031	0.656	n.s	0.177	n.s	0.438
	Total	3.7 ± 0.2	3.4 ± 0.2	3.4 ± 0.3	3.5 ± 0.2	< 0.001	0.137	< 0.001	0.820	< 0.001	0.680	n.s	0.008
Between-sex effects	*p*-value	n.s	< 0.001	< 0.001	< 0.001								
	*d*	0.360	1.104	2.222	1.169								
TJA total score*	Male	4.5 (4–6)	3 (3–4)	3 (2–4)	3 (3–4)	0.015	0.103	0.015	0.721	0.011	1.260	n.s	0.327
	Female	4 (4–5)	4 (3–5)	4.5 (4–5.5)	4 (4–5)	n.s	0.003	n.s	0.127	n.s	0.424	n.s	0.487
	Total	4 (4–5)	4 (3–5)	4 (3–5)	4 (3–5)	n.s	0.032	0.016	0.527	n.s	0.424	n.s	0.110
Between sex effects	*p*-value	n.s	0.031	0.003	0.001								
	*d*	0.637	0.286	0.549	1.501								
TJA number of jumps	Male	16.2 ± 2.3	14.8 ± 1.7	16.4 ± 1.9	15.3 ± 1.9	0.008	0.114	0.032	0.554	n.s	0.061	0.008	0.695
	Female	14.6 ± 1.6	15 ± 1.4	15.8 ± 1.5	15.1 ± 1.5	n.s	0.035	n.s	0.220	n.s	0.558	n.s	0.417
	Total	15.3 ± 2	14.9 ± 1.6	16.1 ± 1.7	15.2 ± 1.8	0.012	0.059	n.s	0.200	n.s	0.295	0.003	0.570
Between sex effects	*p*-value	n.s	n.s	n.s	n.s								
	*d*	0.829	0.131	0.366	0.133								

*Y* years, *CMJ* counter movement jump, *TJA* tuck jump assessment, *DVJ* drop vertical jump, *SD* standard deviation; *ω*_*p*_^*2*^, partial omega squared, *d*, Cohen´s *d*

Effect size limits; *ω*_*p*_^*2*^ ≥ 0.01 = small effect, *ω*_*p*_^*2*^ ≥ 0.06 = medium effect, *ω*_*p*_^*2*^ ≥ 0.14 = large effect; *d* ≥ 0.2 = small effect, *d* ≥ 0.5 = medium effect, *d* ≥ 0.8 = large effect

^*^Median (IQR) shown for TJA total score

Distribution within males; total *n* = 66, 13y *n* = 10, 14y *n* = 43, 15-16y *n* = 13; Distribution within females; total *n* = 49, 13y *n* = 13, 14y *n* = 24, 15-16y *n* = 12

**Table 4:** Age and sex differences in the drop vertical jump test

			Age groups												Between age groups effects	
			13y			14y			15–16y			Total				
			n	% of 13y	% of DVJ score	*n*	% of 14y	% of DVJ score	*n*	% of 15–16 y	% of DVJ score	*n*	% of all ages	% of DVJ score	*p*-value	Cramer´s V ^1^
DVJ	Male	Good control	7	70%	29%	15	35%	63%	2	15%	8%	24	36%	100%	0.006	0.336
		Reduced control	2	20%	7%	17	40%	57%	11	85%	37%	30	45%	100%		
		Poor control	1	10%	8%	11	26%	92%	0	0%	0%	12	18%	100%		
		Total	10	100%	15%	43	100%	65%	13	100%	20%	66	100%	100%		
	Female	Good control	0	0%	0%	4	17%	44%	5	42%	56%	9	18%	100%	n.s	0.287
		Reduced control	10	77%	36%	14	58%	50%	4	33%	14%	28	57%	100%		
		Poor control	3	23%	25%	6	25%	50%	3	25%	25%	12	24%	100%		
		Total	13	100%	27%	24	100%	49%	12	100%	24%	49	100%	100%		
	Total	Good control	7	30%	21%	19	28%	58%	7	28%	21%	33	29%	100%	n.s	0.103
		Reduced control	12	52%	21%	31	46%	53%	15	60%	26%	58	50%	100%		
		Poor control	4	17%	17%	17	25%	71%	3	12%	13%	24	21%	100%		
		Total	23	100%	20%	67	100%	58%	25	100%	22%	115	100%	100%		
Between-sex effects		*p*-value	< 0.001			n.s			0.013			n.s				
		Cramer´s V ^2^	0.757			0.211			0.549			0.197				

*Y* Years, *DVJ* drop vertical jump

1. Effect size limits for Cramer´s V with 4 degrees of freedom; ≥ 0.05 = small effect, ≥ 0.15 = medium effect, ≥ 0.25 = large effect

2. Effect size limits for Cramer´s V with 2 degrees of freedom; ≥ 0.07 = small effect, ≥ 0.21 = medium effect, ≥ 0.35 = large effect

## Discussion

The main findings of the present study were that sprint performance was moderately to strongly correlated with agility and jump performance, and there were weak to moderate correlations between performance tests and TJA, while DVJ did not correlate with the other tests. Boys performed better than girls in performance tests and TJA, and in boys there was an age effect where older players performed better. In contrast, no clear age effect was evident in girls.

Different performance tests were chosen to measure different aspects of football relevant performance that may be motivating for the players. Jump performance is a functional measure of power in football players [39] and jump height is related to team success [1]. Assessment of sprint performance was deemed relevant since sprinting often precedes a goal in football [10]. Sprint performance has been shown to be correlated to lower-body muscle strength in youth male football players [5]. Strong correlations were found between 10- and 20-m sprint tests, in line with earlier research [21]. The correlation between the different performance tests means that a faster time on the agility or the sprint tests correlates with greater explosive jump and endurance jump capacity. The percentage of variability (*r*^2^) in explosive jump and endurance jump capacity explained by the performance in the agility and sprint tests were 38–49% and 36–60%, respectively. Despite the different characteristics of the tests, almost all performance tests showed moderate to strong correlations. The strongest correlations were seen between sprint and agility tests, which is expected since these tests measure similar physical abilities. Sprint and jump performance was also strongly correlated, which supports previous data on youth male football players [5] and on male and female collegiate football players [20]. Acceleration and speed during 505 agility test was only weakly correlated to side hop assessing jump endurance in the frontal plane. In the present study, neuromuscular control assessed during TJA showed weak to moderate correlations with the performance tests. This is reasonable since TJA is a maximum effort test during 10 s, which requires power and endurance similar to the performance tests. In contrast, neuromuscular control assessed during DVJ was not correlated with any other test. This suggests that TJA and DVJ assess different aspects of neuromuscular control. The finding is in line with previous studies that reported differences in assessment of frontal plane kinematics [19] and no correlation between the DVJ and TJA tests [2].

Boys performed better than girls in performance tests and TJA, which is expected and may be explained by sex differences in physiological characteristics. In boys there was an age effect where older players performed better, while no clear age effect was evident in girls. Age-related differences in performance in male youth football players are probably related to maturation [21]. Similar age-related differences in performance has been reported in female football players, although that cohort included a wider age range from 12 to 21 years of age, with the greatest differences between the youngest and the oldest players [40]. The fact that older female players did not perform better compared to their younger counterparts in the present study could possibly be explained by athletic awkwardness caused by adolescent growth spurt during puberty. For instance, a rapid increase in limb length might negatively affect performance and neuromuscular control. However, the fact that girls did not display age-related differences in performance as their male counterparts might also be related to differences in training quality and quantity between sexes.

There were contradictory results on the two tests assessing neuromuscular control, where TJA revealed reduced neuromuscular control in girls compared to boys, but there was no sex difference in DVJ. The DVJ and TJA seem to test different aspects of neuromuscular control and may be used complementary. However, the ecological validity of assessing DVJ for football players has been questioned [36], which is supported be the finding in the present study where DVJ did not correlate to the other tests assessing football relevant performance. Repeated jumping tests such as TJA may better represent the ability of the neuromuscular system to provide adequate stabilization and force attenuation throughout the task [36]. However, the accuracy of identification of risk factors is questioned [35]. Both DVJ and TJA are based on subjective assessments, which has shown to be reliable [15, 28], though the assessment of each athlete is to some extent rater dependent. The subjective assessments of DVJ and TJA have been shown to be mainly concordant but vary between raters and assessments. (Lindblom et al., unpublished data). The uncertainty in the assessments needs to be taken into consideration when interpreting the results of these tests and in clinical practice. The validity of neuromuscular control test to predict sport injuries remains unclear [23], and a key question is determination of cut-off values that separate high-risk and low-risk individuals.

Strengths with the present study design were the utilization of field tests, commonly used in football players, together with standardized instructions and adequate technical equipment. Players performed a structured warm-up before testing and were allowed practice trials before tests and sufficient rest between tests. Subjective assessments of neuromuscular control tests were performed by an experienced sport physiotherapist.

Study limitations involve small samples when comparing different age groups split by sex, and there were unequal distributions in age groups in boys and girls; however, differences were not significant. The test battery included nine different tests to assess different aspects of performance and neuromuscular control, but specific tests of muscle strength and balance were not included. Assessment of adolescents entail special challenges since young athletes might display greater heterogeneity in performance of tests due to large variations in physical maturity, fitness and neuromuscular control compared to adults and elite athletes [34]. Some players had difficulties to correctly perform tests that involved high demands on coordination, especially the agility t-test. Further, it cannot be guaranteed that players performed the tests with maximum effort. To standardize the test procedure all players were given the same instructions and equal amount of encouragement from the test leaders. Players in all teams were also encouraged to motivate each other to perform their best at the tests. Testing was carried out at the start of the second half of the competitive season after the school summer break. The results may not be valid for other time periods of the season, as the time of season may affect the performance [8]. Finally, this study included uninjured youth football players and results may not be generalizable to older players, or players who return to football after an injury.

The present data can be used by coaches and medical professionals when deciding on which tests to include in a test battery for football players. The performance tests and neuromuscular control tests assessed in the present study may be used to determine individual player strengths and limitations and evaluate responses to a training regimen or to monitor development during maturation. The results of the tests may be compared among players within the team or with normative data. The present cohort had symmetrical limb performance and, therefore, data for both legs were used integrated in the analyses. However, limb asymmetry might be apparent in football players and could be evaluated in for instance the return to play after injury [36].

## Conclusion

Sprint performance was moderately to strongly correlated with agility and jump performance, and performance tests were weakly to moderately correlated to TJA, while DVJ did not correlate with the other tests. Boys performed better than girls on all performance tests and in TJA. Age effect on test performance was evident in boys but not in girls.

### Electronic supplementary material

Below is the link to the electronic supplementary material.

Supplementary material 1 (DOCX 31 kb)

## Data Availability

Deidentified data are available from the authors upon reasonable request.

## Appendix

Online appendix. See Table 5.

**Table 5:** Description of the included performance tests and neuromuscular control tests

Test	Test description	No. of practice trials	No. of test trials	
Agility tests				
	Agility t-test (s)	Assessment of change of direction agility. The player ran 10 m forwards towards a cone, side shuffled 5 m to the left, touched a cone with the left hand, side shuffled 10 m to the right and touched a cone with the right hand and then side shuffled 5 m to the left and touched the middle cone with the left hand before sprinting 10 m backwards to the starting position. Recording was done using timing gates positioned at the start and finish line	≥ 2	2^a^
	505 agility test (s)	Assessment of acceleration and speed of making a 180 degree turn [9]. The player sprinted 15 m forwards, through timing gates positioned after 10 m, made a 180 degree turn at the 15 m line and sprinted 5 m back through the same timing gates again	≥ 2	2^a^
Jump tests				
	Single-leg hop for distance (cm)	Assessment of horisontal hop performance. The player started the test standing on the right leg and jumped as far as possible and landed on the same leg. Hands were kept on the back during the test	≥ 3	3^a^
	Side hop (*n*)	Assessment of jump endurance. The player stood on one leg with the hands on the back and jumped as many times as possible for 30 s between two tape markings 40 cm apart [14]. Number of approved jumps per leg was calculated later using the films	Free	1
	Countermovement jump (cm)	Assessment of vertical jump performance. The player quickly bent the knees and then immediately jumped upwards, attempting to maximize jump height. The test was performed with the hands on the hips. An infrared contact mat was used for analysis of jump height	≥ 2	3^a^
Sprint tests				
	10 and 20 m sprint (s)	Assessment of sprint performance. Timing gates were placed at the start, at 10 m and at 20 m.The player stood approximately 30 cm behind the timing gates and started the test upon the test leader’s command, the test time started when the infrared light was crossed	1	2^a^
Neuromuscular control tests				
	Tuck jump assessment	Assessment of neuromuscular control. The player jumped repeatedly for 10 s and attempted to lift the knees to hip level (parallel to the ground) during the jump and start a new jump immediately upon landing. Jump and landing technique was assessed according to ten criteria [25, 27]. A dichotomized grading scale [15] was used	Free	1^b^
	Drop vertical jump	Assessment of neuromuscular control based on the test described by Hewett et al. [16]. The player stood on a 30 cm high and 50 cm wide box with the feet 35 cm separated, dropped down from the box and immediately made a maximum vertical jump and raised both arms to try to reach an overhead target positioned 2.6 m above [11]. The first landing, i.e. the drop from the box, was used for analysis [16]. The frontal knee control was assessed according to a graded scoring scale from 0 to 2 with predefined criteria, e.g. knee alignment and/or presence of valgus and/or medio-lateral movement of one or two knees during the jump (with 0 representing good control, 1 representing reduced control and 2 representing poor control) [28, 38]	Free	3^b^^c^

^a^ The best trial was used for analysis

^b^Trials were filmed and assessed later by an experienced sport physiotherapist. The films were scrutinized as many times as necessary in both real-time and slow-motion

^c^The trial representing the worst technique was used for analysis
