# Supplementary material for: Performance on sprint, agility and jump tests have moderate to strong correlations in youth football players but performance tests are weakly correlated to neuromuscular control tests
Source: Knee Surg Sports Traumatol Arthrosc. 2020 Oct 8;29(5):1659–69. doi: 10.1007/s00167-020-06302-z (PMC8038985; doi:10.1007/s00167-020-06302-z)
Supplement: Supplementary file 1 — Supplementary material 1 (DOCX 31 kb) [file 167_2020_6302_MOESM1_ESM.docx]

Online appendix. Description of the included performance tests and neuromuscular control tests

| Test | | Test description | No of practice trials | No of test trials |
| --- | --- | --- | --- | --- |
| Agility tests | |  |  |  |
|  | Agility t-test (s) | Assessment of change of direction agility. The player ran 10 m forwards towards a cone, side shuffled 5 m to the left, touched a cone with the left hand, side shuffled 10 m to the right and touched a cone with the right hand and then side shuffled 5 m to the left and touched the middle cone with the left hand before sprinting 10 m backwards to the starting position. Recording was done using timing gates positioned at the start and finish line. | ≥2 | 2^a^ |
|  | 505 agility test (s) | Assessment of acceleration and speed of making a 180 degree turn [9]. The player sprinted 15 m forwards, through timing gates positioned after 10 m, made a 180 degree turn at the 15 m line and sprinted 5 m back through the same timing gates again. | ≥2 | 2^a^ |
| Jump tests | |  |  |  |
|  | Single-leg hop for distance (cm) | Assessment of horisontal hop performance. The player started the test standing on the right leg and jumped as far as possible and landed on the same leg. Hands were kept on the back during the test. | ≥3 | 3^a^ |
|  | Side hop (n) | Assessment of jump endurance. The player stood on one leg with the hands on the back and jumped as many times as possible for 30 seconds between two tape markings 40 cm apart[14]. Number of approved jumps per leg was calculated later using the films. | Free | 1 |
|  | Countermovement jump (cm) | Assessment of vertical jump performance. The player quickly bent the knees and then immediately jumped upwards, attempting to maximize jump height. The test was performed with the hands on the hips. An infrared contact mat was used for analysis of jump height. | ≥2 | 3^a^ |
| Sprint tests | |  |  |  |
|  | 10 and 20 m sprint (s) | Assessment of sprint performance. Timing gates were placed at the start, at 10 m and at 20 m.The player stood approximately 30 cm behind the timing gates and started the test upon the test leader’s command, the test time started when the infrared light was crossed. | 1 | 2 ^a^ |
| Neuromuscular control tests | | |  |  |
|  | Tuck jump assessment | Assessment of neuromuscular control. The player jumped repeatedly for 10 seconds and attempted to lift the knees to hip level (parallel to the ground) during the jump and start a new jump immediately upon landing. Jump and landing technique was assessed according to ten criteria [25, 27]. A dichotomized grading scale [15] was used. | Free | 1^b^ |
|  | Drop vertical jump | Assessment of neuromuscular control based on the test described by Hewett et al [16]. The player stood on a 30 cm high and 50 cm wide box with the feet 35 cm separated, dropped down from the box and immediately made a maximum vertical jump and raised both arms to try to reach an overhead target positioned 2.6 m above [11]. The first landing, i.e. the drop from the box, was used for analysis [16]. The frontal knee control was assessed according to a graded scoring scale from 0 to 2 with predefined criteria, e.g. knee alignment and/or presence of valgus and/or medio-lateral movement of one or two knees during the jump (with 0 representing good control, 1 representing reduced control and 2 representing poor control) [28, 38]. | Free | 3^bc^ |

^a^ The best trial was used for analysis.

^b^Trials were filmed and assessed later by an experienced sport physiotherapist. The films were scrutinized as many times as necessary in both real-time and slow-motion.

^c^The trial representing the worst technique was used for analysis.
